# Association of Genes implicated in primary angle-closure Glaucoma and the ocular biometric parameters of anterior chamber depth and axial length in a northern Chinese population

**DOI:** 10.1186/s12886-018-0934-8

**Published:** 2018-10-22

**Authors:** Shaolin Wang, Wenjuan Zhuang, Jianqing Ma, Manyun Xu, Shunyu Piao, Juan Hao, Wen Zhang, Hao Chi, Zhongqi Xue, Shaoping Ha

**Affiliations:** 1 People’s Hospital of Ningxia Hui Autonomous Region, Ningxia Eye Hospital (First Affiliated Hospital of Northwest University For Nationalities), 936 Huang He East Road, Yinchuan, 750011 China; 2 Department of Ophthalmology, Wuzhong People’s Hospital, 430 Wen Wei Road, Wuzhong, 751100 China; 3 Clinical Medical College of Ningxia Medical University, 1160 Shengli Street, Yinchuan, 750001 China; 4 Department of Ophthalmology, Taiyuan Central Hospital, 1 Dong San Dao Lane, Jie Fang North Road, Taiyuan, 030000 China; 5 Shandong Academy of Medical Sciences, Jinan University, 18877 Jing Shi Road, Li Xia District, Jinan, 250000 China

**Keywords:** *ZNRF3*, *HGF*, *MERP*, PACG, ACD, AL, Single nucleotide polymorphisms

## Abstract

**Background:**

The membrane frizzled-related protein (*MFRP*) gene is involved in axial length (AL) regulation and *MFRP* mutations cause nanophthalmos; also, the hepatocyte growth factor (*HGF*) gene is reported to result in morphologic changes of the anterior segment and abnormal aqueous regulation that increases the risk of primary angle-closure glaucoma (PACG), while the zinc ring finger 3 (Z*NRF3*) gene is associated with AL. The present study investigated the association of single nucleotide polymorphisms (SNPs) in *ZNRF3*, *HGF* and *MFRP* with PACG in a northern Chinese population, as well as the association of these SNPs with the ocular biometric parameters of anterior chamber depth (ACD) and AL.

**Methods:**

A total of 500 PACG patients and 720 controls were recruited. All individuals were genotyped for 12 SNPs in three genes (rs7290117, rs2179129, rs4823006 and rs3178915 in *ZNRF3*; rs5745718, rs12536657, rs12540393, rs17427817 and rs3735520 in *HGF*, rs2510143, rs36015759 and rs3814762 in *MFRP*) using an improved multiplex ligation detection reaction (iMLDR) technique. Genotypic distribution was analyzed for Hardy-Weinberg equilibrium. Differences in the allelic and genotypic frequencies were evaluated and adjusted by age and sex. Linkage disequilibrium (LD) patterns were tested and haplotype analysis was conducted by a logistic regression model. Generalized estimation equation (GEE) analysis was conducted using SPSS for primary association testing between genotypes and ocular biometric parameters. Bonferroni corrections for multiple comparisons were performed, and the statistical power was calculated by power and sample size calculations.

**Results:**

The rs7290117 SNP in *ZNRF3* was significantly associated with the AL, with a *p*-value of 0.002. We did not observe any significant associations between the SNPs and PACG or ACD. In a stratification analysis by ethnicity, rs12540393 and rs17427817 in *HGF* showed a nominal association with PACG in the Hui cohort, although significance was lost after correction.

**Conclusions:**

The present study suggests rs7290117 in *ZNRF3* may be involved in the regulation of AL, though our results do not support a contribution of the SNPs we tested in *ZNRF3*, *HGF* and *MFRP* to PACG in northern Chinese people. Further studies in a larger population are warranted to confirm this conclusion.

## Background

Primary angle-closure glaucoma (PACG) is a subtype of glaucoma, characterized by appositional approximation or contact between the iris and trabecular meshwork [1] and is considered to be the most common cause of bilateral glaucoma blindness worldwide [2]. Epidemiological studies have revealed that most PACG cases are in Asia [3], especially in China [4]. PACG has been recognized to be a multifactorial disease, and obvious racial differences [5] and family aggregation [6] have been confirmed in its prevalence, which suggests that genetic factors may play an important role in its pathogenesis. Up until now, 2 genome-wide association studies (GWAS) on PACG have been conducted and 8 genetic loci showed strong associations with the disease [7, 8]. In another GWAS on anterior chamber depth (ACD), the rs1401999 locus in the *ABCC5* gene was also found to be associated with PACG [9]. However, these genes only partly explain the genetic predisposition to PACG.

Furthermore, the membrane frizzled-related protein (*MFRP*) gene was related to nanophthalmos [10] while the hepatocyte growth factor (*HGF*) gene was reported to be associated with hyperopia [11], and both nanophthalmos and hyperopia are important risk factors for PACG [10, 12]. Meanwhile, in previous studies, the association between *HGF* and PACG has been evaluated in two different populations by the candidate gene approach [13, 14] and validated in a meta-analysis by Rong et al., although sample sizes were relatively small compared with the GWAS, which might lead to false-positive signals [15]. In addition, the zinc ring finger 3 (Z*NRF3*) gene was confirmed to be associated with axial length (AL) in a GWAS meta-analysis [16]. Consequently, the aim of this study was to evaluate the association of the three susceptibility genes with PACG in a northern Chinese population. In essence, we were interested in the association between these single nucleotide polymorphisms (SNPs) and the ocular biometric parameters of ACD and AL.

## Methods

### Subjects

A total of 500 cases with PACG and 720 ethnic-matched controls were recruited from the northern regions of China. The study was approved by the local hospital’s ethics committee and met the tenets of the Declaration of Helsinki. Informed consent was obtained from all subjects prior to the study. Comprehensive ophthalmic examinations for each participant were performed, including best-corrected visual acuity, intraocular pressure (IOP) measurement, slit lamp biomicroscopy, fundus photography, visual field, gonioscopy and ultrasound biomicroscopy. ACD and AL were measured by IOL Master. Five readings were obtained and the mean value was used for further statistical analysis. PACG patients were diagnosed by fulfilling all of the following criteria: IOP of more than 21 mmHg; the presence of at least two quadrants of closed angle in which the trabecular meshwork was not visible on gonioscopy; the presence of glaucomatous damage to the optic nerve with a cup-to-disc ratio ≥ 0.7 and peripheral visual loss, in accord with the International Society of Geographical and Epidemiological Ophthalmology (ISGEO) [17]. The controls were required to have none of the above characteristics and have open angles verified by gonioscopy, no known family history of glaucoma and previous glaucomatous or cataractous operations, no other ophthalmic diseases besides mild senile cataracts. Participants with secondary angle-closure glaucoma caused by trauma, uveitis, or neovascularization, were excluded.

### DNA extraction

Peripheral venous blood samples were collected from all participants and genomic DNA was isolated from the blood samples utilizing the Simgen DNA Blood Mini Kit (Simgen, Hangzhou, China) in accordance with the manufacturer’s protocol. The extracted DNA was eluted in TE buffer (10 mM Tris-HCl, 0.5 mM EDTA, pH 9.0) and then stored at − 80° until use after the A260/A280 optical density was measured with Nanodrop2000 (Thermo Fisher Scientific Inc., Wilmington, DE, USA).

### SNP selection and genotyping

Since associations or possible associations between our target genes and PACG were reported in previous studies [13, 14, 18–20], a total of 12 SNPs were chosen as candidates. They were rs7290117, rs2179129, rs4823006 and rs3178915 in *ZNRF3*; rs5745718, rs12536657, rs12540393, rs17427817 and rs3735520 in *HGF;* rs2510143, rs36015759 and rs3814762 in *MFRP*. All SNPs were genotyped by Genesky Biotechnologies Inc. (Shanghai, China) using an improved multiplex ligation detection reaction (iMLDR) technique.

### Statistical analysis

Demographic differences between the cases and controls were performed using the SPSS software (version 17.5: SPSS Science, Chicago, IL), the differences in sex and ethnicity were assessed by the χ2 test and the differences in age were assessed by T test. Each SNP was appraised for compliance with Hardy-Weinberg equilibrium (HWE) using the χ2 test. The genetic association analyses as well as the meta-analysis were conducted using PLINK (version 1.07; http://zzz.bwh.harvard.edu/plink/index.shtml, in the public domain). Allelic and genotypic frequency differences of a given SNP between the PACG patients and the controls were evaluated and adjusted by age and sex using a logistic regression model. Meanwhile, the adjusted odds ratios (ORs) and the corresponding 95% confidence intervals (CIs) for associations were also presented. Linkage disequilibrium (LD) patterns were tested with Haploview 4.2 software (Daly Lab at the Broad Institute, Cambridge, MA), and haplotype analysis was also conducted by a logistic regression model and adjusted for age and sex. Generalized estimation equation (GEE) analysis with an unstructured working correlation matrix modeling for a trend-per-copy effect on the minor allele (coding 0 for the wild-type genotype, 1 for heterozygous genotype, and 2 for homozygous genotype for the minor allele) was performed using SPSS for association testing between genotypes and ocular biometric parameters. In this analysis, the genotypes were treated as covariates, ACD and AL were control variables of each other, and age and sex were adjusted. Bonferroni correction was performed if a positive association (a *p*-value of less than 0.05) was found in the initial analysis. The statistical power was calculated by the Power and Sample Size Calculation (PS; version 3.1.232).

## Results

This study comprised 500 PACG patients (147 males and 353 females; 93 Hui and 407 Han) and 720 control subjects (332 males and 388 females; 129 Hui and 591 Han) from the northern regions of China. There were no significant differences in ethnicity between cases and controls. However, the control subjects were significantly older (mean age 71.82 ± 7.2 years vs. 63.77 ± 9.576 years; *p* = 0.000, which was an intentional design for this age-related disease) and included less women (53.9% vs. 70.6%; p = 0.000) than the case group (Table 1).

**Table 1:** Demographic characteristics of PACG cases and controls

	Cases	Controls	*P*
Number	500	720	
Age, y (Mean ± SD)	63.77 ± 9.576	71.82 ± 7.2	0.000#
Sex, *n* (%)			0.000*
Male	147 (29.4)	332 (46.1)	
Female	353 (70.6)	388 (53.9)	
Ethnicity, *n* (%)			0.761*
Han	407 (81.4)	591 (82.1)	
Hui	93 (18.6)	129 (17.9)	

#The *p*-value was tested by T-test

*The *p*-value was assessed by χ2 test

**Fig. 1:**
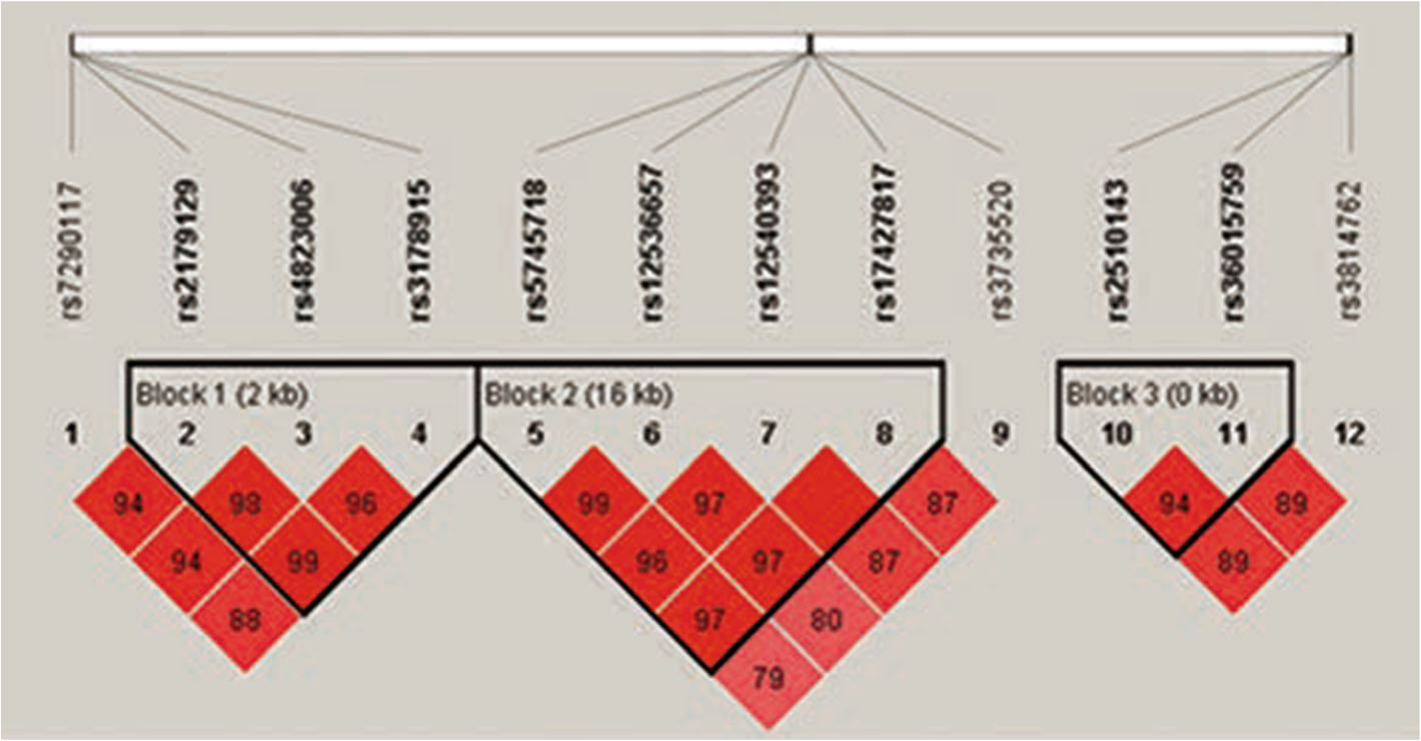
Three Haplotype Blocks of the 12 Target SNPs. Nine target SNPs are presented to the three haplotype blocks in HapMap CHB cohort combined of PACG and control, which were determined by the Haploview 4.2 program. Darker shades suggested higher linkage disequilibrium

The genotyping call rates for the 12 SNPs in both case and control groups were more than 99% and their allele distributions were within HWE (*P* > 0.05) (Table 2). The distributions of the allele and genotype frequencies of all SNPs were not significantly different between PACG patients and control subjects. Haplotype analysis was also performed and none of the common haplotypes showed any significant differences between PACG patients and control subjects (Fig. 1, Table 3). Meanwhile subanalysis was also performed within the Hui PACG cases versus Hui controls and Han PACG cases versus Han controls since the participants recruited included two peoples. Rs12540393 and rs17427817 in *HGF,* with the same *p*-value of 0.019*,* were associated with PACG in the Hui cohort after correction for age and sex using logistic regression and the frequencies of the minor C allele of rs12540393 as well as rs17427817 were less in the PACG group than in the control group. However, the significance was lost after Bonferroni correction. None of the remaining SNPs and haplotypes were associated with PACG in either the Hui or Han cohort. We amalgamated the results of the separate analyses of the two different ethnicities, and the meta-analysis *p*-values were almost the same as the initial overall analysis (Table 4).

**Table 2:** Association results of target SNPs with PACG after adjustment for age and sex

GENE	SNP	CHR	BP	Minor allele	Genotype (AA/AB/BB)^a^		MAF		HWE-*p*		OR (95% CI)	*P*
					Case	Control	Case	Control	Case	Control		
*ZNRF3*	rs7290117	22	29,450,856	T	427/72/1	632/84/4	0.074	0.064	0.5056	0.524	1.306(0.9007~ 1.894)	0.159
*ZNRF3*	rs2179129	22	29,450,923	G	179/238/83	236/347/137	0.404	0.431	0.7813	0.6487	0.9095 (0.7571~ 1.092)	0.3105
*ZNRF3*	rs4823006	22	29,451,671	G	130/252/118	200/381/139	0.488	0.458	0.9287	0.08442	1.166 (0.9671~ 1.407)	0.1073
*ZNRF3*	rs3178915	22	29,453,027	A	168/243/88	252/358/108	0.419	0.400	1	0.3128	1.085 (0.8992~ 1.309)	0.3954
*HGF*	rs5745718	7	81,347,548	T	366/124/10	537/172/11	0.144	0.134	1	0.6314	0.9475 (0.7256~ 1.237)	0.6924
*HGF*	rs12536657	7	81,350,208	A	363/126/10	534/174/11	0.146	0.136	1	0.5287	0.9514 (0.7292~ 1.241)	0.7132
*HGF*	rs12540393	7	81,364,187	C	342/143/14	513/191/16	0.171	0.155	1	0.8865	0.9266 (0.7216~ 1.19)	0.5503
*HGF*	rs17427817	7	81,364,435	C	342/144/14	512/192/16	0.172	0.155	0.8764	0.7774	0.9249 (0.7203~ 1.188)	0.5405
*HGF*	rs3735520	7	81,400,939	A	139/258/103	227/349/144	0.464	0.442	0.4199	0.6505	1.191 (0.9897~ 1.433)	0.06434
*MFRP*	rs2510143	11	119,216,231	A	372/119/9	515/186/19	0.137	0.156	1	0.6699	0.8559 (0.6603~ 1.109)	0.2396
*MFRP*	rs36015759	11	119,216,279	A	289/178/33	425/252/43	0.244	0.235	0.4665	0.4694	1.085 (0.8794~ 1.338)	0.4478
*MFRP*	rs3814762	11	119,216,504	T	328/151/21	479/211/29	0.193	0.187	0.4746	0.3285	0.946 (0.7259~ 1.189)	0.6337

^a^A represents the wild-type allele, B represents the minor allele; *CHR* chromosome, *BP* base pair position, *MAF* minor allele frequency, *HWE-p* the *p*-value of Hardy-Weinburg equilibrium, *OR* odds ratio, *CI* confidence interval

*P-*value, OR, and CI were calculated with a logistic regression model by adjusting for age and sex

**Table 3:** Haplotype analysis of the target genes in PACG and control cohorts

Block	SNPS	Haplotype	Freq of cases (%)	Freq of controls (%)	OR	*P-value*
Block 1	rs2179129, rs4823006, rs3178915	AGA	41.77	38.74	1.15	0.153
		AGG	6.92	6.54	1.15	0.476
		GAG	40.29	42.48	0.924	0.403
		AAG	10.69	10.48	0.96	0.785
Block 2	rs5745718, rs12536657, rs12540393, rs17427817	TACC	13.81	13.09	0.926	0.575
		GGCC	3.02	2.02	0.961	0.895
		GGTG	82.25	84.26	1.05	0.687
Block 3	rs2510143, rs36015759	GA	24.18	23.35	1.1	0.396
		AG	13.48	15.41	0.857	0.251
		GG	62.12	61.05	1.01	0.907

OR and *P*-value were calculated with the logistic regression model by adjusting for age and sex

**Table 4:** Associations for target SNPs between cases and controls in different ethnicities as well as the meta-analysis results

SNP	MAF-case		MAF-control		OR (95% CI)		*p*		*P-meta* ^a^	I^2^	*P-het*
	HUI	HAN	HUI	HAN	HUI	HAN	HUI	HAN			
rs7290117	0.06452	0.07617	0.05118	0.06684	2.103 (0.8305~ 5.237)	1.151 (0.7692~ 1.723)	0.1169	0.4937	0.184	21.57	0.2588
rs2179129	0.4409	0.3956	0.4488	0.4272	0.877 (0.5951~ 1.293)	0.9251 (0.7511~ 1.139)	0.5073	0.4638	0.3643	0	0.7915
rs4823006	0.457	0.4951	0.4882	0.4509	0.9995 (0.667~ 1.498)	1.194 (0.9665~ 1.475)	0.998	0.1002	0.1359	0	0.4326
rs3178915	0.3925	0.4261	0.4252	0.3947	0.9102 (0.5945~ 1.394)	1.123 (0.9105~ 1.385)	0.6652	0.2783	0.4353	0	0.3858
rs5745718	0.1022	0.1536	0.1732	0.1261	0.573 (0.3114~ 1.054)	1.061 (0.784~ 1.435)	0.07339	0.7022	0.5334	68.41	0.0752
rs12536657	0.1022	0.1564	0.1732	0.128	0.5612 (0.3048~ 1.033)	1.074 (0.7944~ 1.452)	0.06371	0.6423	0.5369	71.26	0.0621
rs12540393	0.1183	0.1835	0.2087	0.143	0.5067 (0.2872~ 0.8939)	1.077 (0.8089~ 1.433)	0.01892	0.6131	0.4807	81.32	0.0207
rs17427817	0.1183	0.1843	0.2087	0.1438	0.5067 (0.2872~ 0.8939)	1.069 (0.8035~ 1.421)	0.01892	0.6485	0.4782	81.23	0.021
rs3735520	0.4839	0.4595	0.4094	0.4492	1.375 (0.9151~ 2.065)	1.158 (0.9402~ 1.426)	0.1254	0.1675	0.05236	0	0.4662
rs2510143	0.172	0.129	0.2047	0.1455	0.7626 (0.4533~ 1.283)	0.872 (0.6455~ 1.178)	0.3072	0.3722	0.2059	0	0.6545
rs36015759	0.2581	0.2408	0.1969	0.2437	1.526 (0.938~ 2.481)	1.005 (0.7954~ 1.27)	0.08872	0.9671	0.4457	57.53	0.1249
rs3814762	0.1452	0.2039	0.1693	0.1912	0.7363 (0.412~ 1.316)	0.988 (0.7692~ 1.269)	0.3014	0.9249	0.6413	0	0.3552

*MAF* minor allele frequency, *OR* odds ratio, *CI* confidence interval, *I*^*2*^ measures heterogeneity, *p-het p*-value for heterogeneit;

*P*-value, OR, and CI were calculated with a logistic regression model by adjusting for age and sex

^a^P-meta, *P*-value obtained by meta-analysis, if the I^2^ value was ≥50%, we took the value of random-effects; otherwise, a fixed-effects model was adopted

Furthermore, in association testing between the 12 SNP genotypes and AL and ACD ocular biometric parameters using GEE tests, we found rs7290117 in *ZNRF3* was associated significantly with the AL with a *p*-value of 0.002 (adjusted *p*-value was 0.024), the variant allele of which may have the effect of making the AL shorter (β = − 0.169) (Table 5).

**Table 5:** Association results between the target Loci, AL, and ACD

GENE	SNP	Minor allele	AL (22.92 ± 0.891; 20.01~ 25.51)^a^			ACD (2.74 ± 0.474; 0.25~ 4.51)^a^		
			*β*	SE	*P*	*β*	SE	*P*
*ZNRF3*	rs7290117	T	−0.169	0.055	0.002	−0.008	0.0312	0.808
*ZNRF3*	rs2179129	G	0.003	0.0313	0.925	0.001	0.0157	0.925
*ZNRF3*	rs4823006	G	−0.023	0.0317	0.461	0.007	0.0159	0.666
*ZNRF3*	rs3178915	A	0.025	0.0313	0.42	0.005	0.0161	0.74
*HGF*	rs5745718	T	−0.005	0.0417	0.912	0.011	0.0243	0.64
*HGF*	rs12536657	A	0.001	0.0415	0.978	0.01	0.0243	0.69
*HGF*	rs12540393	C	−0.027	0.0394	0.495	0.012	0.023	0.594
*HGF*	rs17427817	C	−0.026	0.0393	0.516	0.011	0.023	0.647
*HGF*	rs3735520	A	−0.018	0.0309	0.568	−0.024	0.0157	0.127
*MFRP*	rs2510143	A	−0.016	0.0432	0.714	0.012	0.0218	0.581
*MFRP*	rs36015759	A	0.04	0.0339	0.234	0.009	0.0195	0.654
*MFRP*	rs3814762	T	−0.014	0.0362	0.703	0.002	0.0202	0.918

^a^Numbers in parentheses indicate the Mean ± SD and the range of measured values for AL or *ACD β,* per-allele effect in ACD/AL, *SE* standard error for ascertainment of *β*, *P, P*-value for association adjusting for age and sex

The power varies between the 12 SNPs due to the difference of their minor allele frequency (MAF). Therefore, assuming an allelic OR of 1.5, our sample size provides more than 95% statistical power to detect a significant association at an α level of 0.05 with the exception of the SNP rs7290117, which has 77% statistical power to detect a significant association in the same conditions.

## Discussion

PACG is a multifactorial disease, and both genetic and environmental factors are significant to its progression [1]. Candidate gene approaches have been used to explore the genetic architecture of glaucoma and some possible susceptibility genes have been reported. In the present study, we chose three genes that were previously reported as having an association with regulation of AL [10, 16] or hyperopia [11] to evaluate the association between these genes and PACG in a northern Chinese cohort. Consequently, we did not observe any association between the three target genes and PACG. However, rs7290117 in *ZNRF3* was validated to be significantly associated with the AL by the GEE method [21, 22], which is suitable for statistical analysis of correlated data since binocular biometric parameters can better reflect the genetic characteristics. To the best of our knowledge, this is the first study to investigate the association of the AL-related gene *ZNRF3* with PACG.

PACG patients have similar anatomical features, such as shallow anterior chambers and short AL [2]. Recently, Cheng et al. found rs12321 in *ZNRF3* was associated with AL in a GWAS meta-analysis [16], and proteins encoded by *ZNRF3* are directly involved in the Wnt signaling pathway [23], which is a significant pathway in vertebrate eye development [24]. Shi et al. evaluated the association between *ZNRF3* and primary angle-closure (PAC) in a Chinese cohort and found no association between them [20]. In our study, we also failed to find any association between *ZNRF3* and PACG. Nevertheless, we found rs7290117 in *ZNRF3* was significantly associated with the AL, which is in line with a previous GWAS meta-analysis [16].

The *HGF* gene has been confirmed to be involved in the emmetropization process of the eye and stimulating the growth and migration of many eye tissues [25–27]. A recent study found some SNPs of the *HGF* gene were associated with susceptibility to hyperopia [11]. Several SNPs of the *HGF* gene were also associated with PACG in different populations [13, 14], Awadalla et al. found four SNPs (rs5745718, rs12536657, rs12540393 and rs17427817) in *HGF* were significantly associated with PACG in a case-control study comprised of 106 patients and 204 controls in the Nepalese population [13], Jiang et al. identified two SNPs (rs5745718 and rs1742817) and a haplotype in *HGF* associated with PACG in a case-control study comprised of 238 patients and 287 controls from the east of China [14], and Rong et al. confirmed the association between the SNPs rs5745718 as well as rs1742817 and PACG through a meta-analysis [15]. In our study, we found rs12540393 and rs17427817 in *HGF* showed a nominal association with PACG in the Hui cohort, and the odds ratios of the two SNPs were contrary to previous findings and the Han cohort. Although the significance was lost after Bonferroni correction, to some extent, such results reflected ethnic differences in disease pathogenesis and implied the association of markers was diverse in different ethnic groups. Considering that the small sample size of the Hui cohort in our study is likely to result in false-positive consequences, the relationship between *HGF* and PACG in different populations still needs further study.

*MFRP* is located on human chromosome 11q23.3, and the COOH terminal domain of *MFRP* is known to be related to the Wnt binding cysteine-rich domain of the frizzled family of transmembrane proteins which are receptors for the Wnt signaling pathway [28], a significant pathway in vertebrate eye development [24]. Mutations in *MFRP* were reported to cause autosomal recessive nanophthalmos, which is characterized by short AL, a small corneal diameter, a high lens/eye volume ratio, and a high degree of hyperopia [10]. Therefore, *MFRP* was considered to be a candidate gene for PACG as well as PAC, however, previous studies did not indicate any significant association between *MFRP* and PACG or PAC in different populations [18, 19, 29], similar to our finding.

Moreover, in the present study, we failed to validate any association between the two nanophthalmos or hyperopia-related genes (*MFRP, HGF*) with AL and ACD or between the three target genes and PACG, since nanophthalmos shows the same characteristics as PACG of a short AL and hyperopia is an important phenotype associated with PACG. Our results suggest that the genes associated with a phenotype of a certain disease are not necessarily related to the disease itself since a disease may have many complex phenotypes, with one or some that are not equal to the disease. Exploration of a certain phenotype is only a tiny point in understanding of the disease, but the understanding of many such “tiny points” will eventually produce an objective and comprehensive understanding of the disease, as in existing genetic association studies, where the function of a single susceptibility locus may be tiny and confusing. Therefore, deeper and more extensive research is necessary. Furthermore, this study involved two ethnic groups, which might make the result of the overall analysis lack credibility. However, we performed a meta-analysis of the two different ethnic groups, and the meta-analysis *p*-values were almost the same as the initial overall analysis. This proves that our initial overall analysis results are reliable and an ethnic-matched case-control study design is feasible when it involves two different ethnicities with small sample size.

The limitation of our research is that the SNPs were chosen on the basis of previous studies but did not utilize the tagger program, which is a common method for candidate gene research and often presents different results in different populations. The SNPs selected thus may not completely represent the genes in our cohort. Therefore, tagger SNPs based on our cohort should be selected for more in-depth study based on pathogenesis in the future.

## Conclusions

We conducted a case-control study of 12 SNPs among 500 PACG subjects and 720 ethnic-matched controls using a candidate gene approach. Our results do not support contribution of the SNPs we tested in *ZNRF3*, *HGF* and *MFRP* to PACG in northern Chinese people. However we confirmed the association of rs7290117 in *ZNRF3* with AL which suggests rs7290117 might be involved in the regulation of ocular biometric parameters of AL in PACG. Further studies in a larger population are needed to verify this conclusion.

## Abbreviations

ACD: anterior chamber depth
AL: axial length
CIs: confidence intervals
GEE: Generalized estimation equation
GWAS: genome-wide association studies
*HGF*: hepatocyte growth factor
HWE: Hardy-Weinberg equilibrium
iMLDR: improved multiplex ligation detection reaction
IOP: intraocular pressure
ISGEO: International Society of Geographical and Epidemiological Ophthalmology
LD: Linkage disequilibrium
MAF: minor allele frequency
*MFRP*: membrane frizzled-related protein
OR: odds ratio
PACG: primary angle-closure glaucoma
SNPs: single nucleotide polymorphisms
*ZNRF3*: zinc ring finger 3
